# Ultra-Orthodox women in the job market: What aid them to become healthy and satisfied?

**DOI:** 10.1192/j.eurpsy.2023.1740

**Published:** 2023-07-19

**Authors:** O. Braun-Lewensohn, T. Kalagy, S. Abu-Kaf

**Affiliations:** 1 Conflict Management & Resolution Program; 2 Department of Public Policy & Administration, Ben-Gurion University of the Negev, Beer-Sheva, Israel

## Abstract

**Introduction:**

Culture and ethnicity are crucial to our identity and responsible for our health, values and thereby to our satisfaction from work.

**Objectives:**

To this end, this study focused on the minority groups of ultra-orthodox women in their work sphere and examined differences between women who work within the enclave, women who work both with ultra-Orthodox and other sectors of the Israel society and women who work mainly outside the ultra-Orthodox enclave on the different study variables. Moreover, a model which include main resources [family, community, diversity climate perceptions (in the job environment) and inclusive leadership] as potential explanatory factors of employees’ satisfaction from work and mental health.

**Methods:**

Data were gathered from 304 ultra-Orthodox women who belong to various streams in this society, who were recruited by the Midgam research panel. The participants filled out self-reported questionnaires among which family quality of life, community sense of coherence, diversity climate, inclusive leadership, job satisfaction and mental health. The participants’ age ranged between 19-64 years (M=30.86 SD=8.71).

**Results:**

The explanation of the full model for jobs satisfaction was: 46% of the variance among women within the enclave, 60% among women who work in mixed environment, and 53% among women who work outside the enclave. As for mental health: 22% of the variance among women within the enclave, 17% among women in mixed environment, and 41% among women outside the enclave.

**Conclusions:**

The results are analyzed through the lens of Bronfenbrenner’s ecological theory and show that in traditional societies such as the ultra-Orthodox one, the most important factors for job satisfaction and mental health are family and communal resources.

**Disclosure of Interest:**

None Declared

